# Thermal Insulating Concrete Wall Panel Design for Sustainable Built Environment

**DOI:** 10.1155/2014/279592

**Published:** 2014-08-06

**Authors:** Ao Zhou, Kwun-Wah Wong, Denvid Lau

**Affiliations:** ^1^Department of Architecture and Civil Engineering, City University of Hong Kong, Kowloon, Hong Kong; ^2^Department of Civil and Environmental Engineering, Massachusetts Institute of Technology, Cambridge, MA, USA

## Abstract

Air-conditioning system plays a significant role in providing users a thermally comfortable indoor environment, which is a necessity in modern buildings. In order to save the vast energy consumed by air-conditioning system, the building envelopes in envelope-load dominated buildings should be well designed such that the unwanted heat gain and loss with environment can be minimized. In this paper, a new design of concrete wall panel that enhances thermal insulation of buildings by adding a gypsum layer inside concrete is presented. Experiments have been conducted for monitoring the temperature variation in both proposed sandwich wall panel and conventional concrete wall panel under a heat radiation source. For further understanding the thermal effect of such sandwich wall panel design from building scale, two three-story building models adopting different wall panel designs are constructed for evaluating the temperature distribution of entire buildings using finite element method. Both the experimental and simulation results have shown that the gypsum layer improves the thermal insulation performance by retarding the heat transfer across the building envelopes.

## 1. Introduction

The air-conditioning system is an important component in quite a number of buildings for supplying a thermally comfortable indoor environment to the users, yet it is accompanied with various environmental and energy concerns including global warming and huge energy consumption. The predicted global average surface warming by the end of the 21st century will range from 0.3 to 6.5°C, and such temperature rise will have a direct and enormous negative impact on environment in which people live in [1, 2]. In high temperature area, the outdoor air temperature could reach 35°C during summer. Exterior surfaces of building envelopes including roof and the external wall surfaces could reach up to 60°C or even higher if they are exposed to direct sunlight [3, 4]. There could be a 35°C temperature difference across the building envelopes if a 25°C designed indoor temperature is maintained by an air-conditioning system. Consequently, a large amount of electrical energy is required by the air-conditioning system for maintaining the required indoor temperature. In order to reduce the electricity consumption by the air-conditioning system, a good thermal insulating building envelope is essential to minimize the unwanted heat transfer between the outdoor and indoor environments, especially for envelope-load dominated buildings [5, 6]. In the United States of America, 46.6% of buildings energy was used for space heating or cooling in 2010 [7], which occupies the largest portion of buildings energy, and much effort has been spent by the industries to improve the thermal insulation of the building envelopes and to decrease the heating and cooling loads [8].

**Figure 1:**
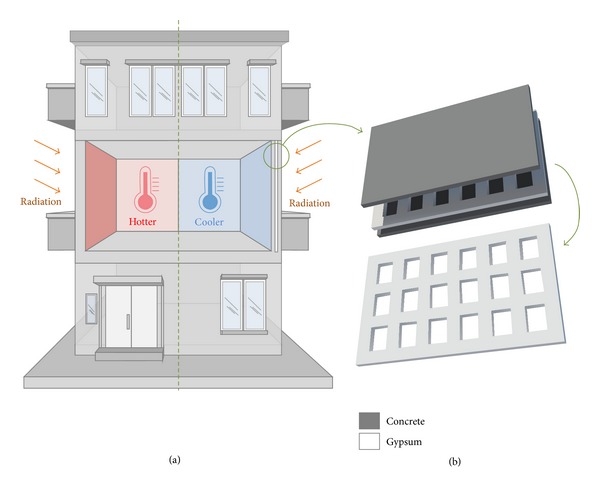
(a) The left part of the building envelopes is constructed by the conventional precast concrete wall panel, while the right part employs the proposed new wall panel design. If there is no air-conditioning system, a building structure adopting the new wall panel will exhibit a lower indoor temperature than that of a conventional concrete building; (b) a schematic diagram of the sandwich concrete/gypsum wall panel which shows the gridded gypsum layer.

A lot of studies were conducted to optimize the performance of building insulation, taking into consideration of building type and orientation, climate condition, building materials, energy cost, efficiency, cost of air-conditioning system, and so forth [9]. It is noticed that an appropriate design of thermal insulation in building envelopes can significantly reduce the amount of electricity (a form of high quality energy) consumed for space heating and cooling and eventually reduce the degradation of energy-quality and caused CO_2_ emissions, which is in line with the concept of sustainable building [10–13]. According to the law of heat transfer [14], the heat flux through a building wall depends on the temperature difference between outdoor and indoor environments, the thermal conductivity of the construction material, and the wall thickness. All these parameters form the basis to characterize the thermal resistance performance of a building [9]. Building materials possess the inertia against the outside temperature fluctuations leading to the hindrance of the thermal equilibrium between the concerned system and the surrounding, which is regarded as thermal mass. Using more concrete in construction can increase the thermal mass of the building, leading to smaller temperature fluctuation of indoor environment. As the insulation thickness in building envelope increases, the loads of heating and cooling of a building decrease. However, such approach is uneconomical and wastes a lot of building spaces. The objective of this paper is to provide a green building design approach that can reduce the energy cost on air-conditioning system, so that the reduction of carbon emission can be achieved. Here, a sandwich concrete/gypsum wall panel design using a composite system concept is proposed, which is economical and is able to achieve a better thermal insulation performance of building envelopes. The sandwich concrete/gypsum wall panel is formed by adding gypsum in the middle of conventional concrete wall so that the new wall panel design consists of three layers, that is, concrete layer, gypsum layer, and concrete layer. Extruded polystyrene concrete sandwich panel is also used in existing industry, where extruded polystyrene is sandwiched by two concrete layers. Compared to the polymer material, the gypsum provides a good thermal mass and the thermal mass of overall wall panel has increased. Furthermore, gypsum is an environmental friendly material, which possesses a low environment impact and provides dependable thermal performance. It is expected that a lower indoor temperature inside a building (probably without any air-conditioning systems) can be achieved using proposed sandwich wall panel design, as shown in Figure 1(a). The proposed sandwich concrete/gypsum wall panel is designed for envelope-load dominated building, such as low-rise residential building, which is greatly affected by outer climatic environment and the internal heat gains are low. In addition, the strategy implemented in this new wall panel is in line with the life cycle assessment (LCA), which can help save a significant amount of building energy and lead to a sustainable development in a built environment [15].

In this research work, both experimental and numerical simulations have been conducted. For the envelope-load dominated building adopting sandwich concrete/gypsum wall panel, convection and radiation are still occurring on concrete surface, which is the same with conventional concrete building. Therefore, the conduction from illuminated concrete surface to unilluminated concrete surface is the primary concern in present study. The thermal conductivities of concrete and gypsum are determined through the experiment, together with the parametric studies. The thermal conductivity, density, specific heat capacity, convective heat transfer coefficient, and surface emissivity of materials are necessary to evaluate the temperature distribution and heat flux in transient heat transfer process of three-story buildings using finite element modeling. It is envisioned that proposed wall panel design can effectively save a significant amount on building energy consumption in terms of electricity spent on air-conditioning system.

## 2. Experimental Materials and Methods

### 2.1. Materials

Two kinds of materials are used in fabricating specimens, namely, concrete and gypsum. A recent experimental study, which investigated the thermal conductivities of different materials used in construction, has shown that concrete possesses the worst thermal resistance when compared with masonry brick and red clay brick [16]. Although the precast concrete is not the best for thermal insulation, it is still one of the most extensively used construction materials in practice because of the following advantages [17, 18]. Firstly, the shape and the dimensions of each precast concrete unit can be standardized with a mass production. Also, comparing to the use of in situ concrete, less supporting formwork is required, which leads to a more economical construction process. Secondly, the quality of precast concrete is generally better and more reliable when compared with that of in situ concrete. Owing to these merits of precast concrete, it is adopted worldwide, and it is expected that the improvement of thermal insulating performance of precast concrete panel will further increase the popularity of precast unit in building construction.

Gypsum has been used as building material from time immemorial. At present, the application of gypsum is still broad in construction industry because of its low cost and the ease of availability. In addition, it is recognized as an environmental friendly material with a low embodied energy [19]. Gypsum (CaSO_4_ *·*2H_2_O) contains water in its chemical composition, in which water can effectively increase its thermal insulation. In fact, the thermal conductivity of gypsum is smaller than that of concrete. It is expected that by adding a gypsum layer into precast concrete, the heat transfer process of the entire precast unit can be effectively retarded.

### 2.2. Test Specimens

**Figure 2:**
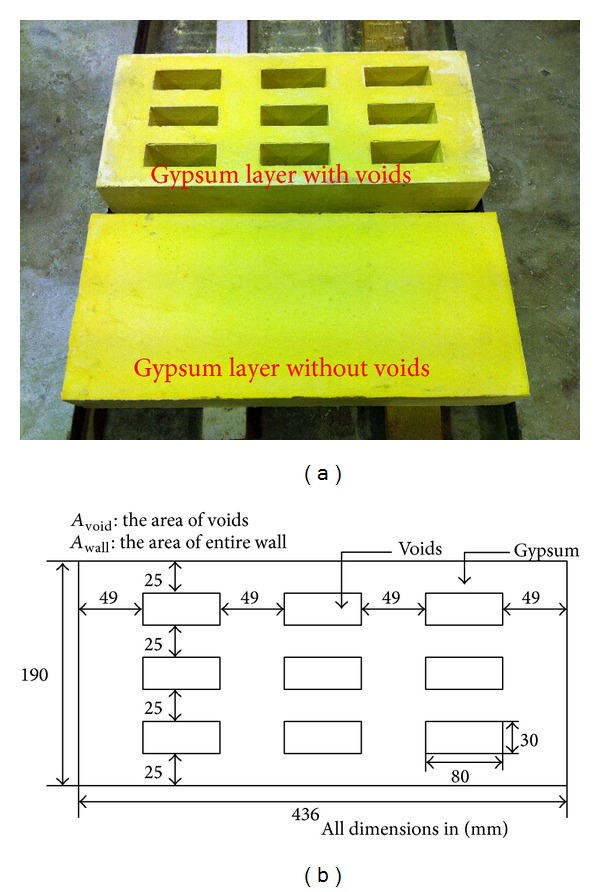
(a) Two types of tested gypsum layers: solid and gridded (with voids); (b) the key dimensions in the gypsum layer with voids.

**Figure 3:**
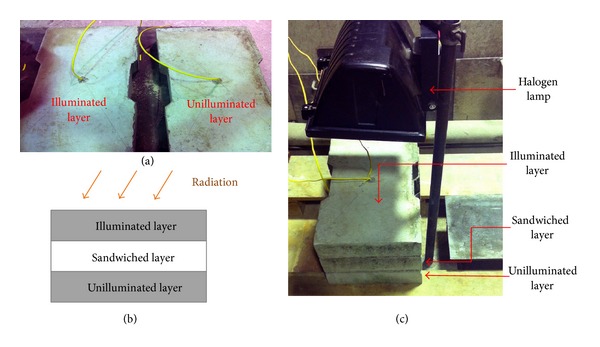
(a) Two concrete layers used as the illuminated and unilluminated layers with a thermocouple embedded in each of them; (b) a schematic diagram showing the specimen and the heat source; (c) the experimental setup.

Compared with the conventional precast concrete wall panel, the new sandwich concrete/gypsum wall panel design contains a gypsum layer inside the precast concrete as shown in Figure 1(b). In order to determine the thermal insulation performance of the sandwich concrete/gypsum wall panel and compare it with the conventional concrete wall, a series of heat transfer tests was performed such that the temperature variation across the wall thickness over time in different specimens could be measured. In addition, an inclusion of air voids in the gypsum layer was investigated experimentally for a comprehensive understanding on the thermal insulation performance of this new wall panel design. It should be mentioned that the strength of the new wall panel still meets the design load criteria by adopting the same structural design approach of conventional precast concrete [20]. Three different types of sandwiched layers were adopted in this experiment, namely, concrete layer, solid gypsum layer, and gypsum layer with voids. Two types of gypsum layers are shown in Figure 2(a) and the dimensions of voids are indicated in Figure 2(b). The voids in the gypsum panel were arranged in a 3 × 3 array and the voids were introduced by placing 9 polystyrene foam cubes in the mold during the casting process, and the polystyrene cubes were removed after the hardening of gypsum. The other solid gypsum panel was also casted using the same mold without the presence of the polystyrene foam cubes. The gypsum layers were then covered (sandwich) by two concrete layers, as shown in Figure 3(a). The nomenclature for each of the specimens is based on its sandwiched layer (written in capital letters), that is, C, G, and GV, where C represents the specimen having a concrete sandwiched layer, G means the specimen having a solid gypsum sandwiched layer, and GV stands for the specimen having a gypsum sandwiched layer with voids. It should be mentioned that the thickness of all layers was 65 mm. Afterwards, the surfaces of all layers were polished to achieve flat and smooth surfaces so that close contact between layers could be obtained. Using this approach, the effect of interface between concrete and gypsum on the heat transfer from illuminated layer to unilluminated layer can be minimized. Figure 3(b) shows the schematic diagram of the entire testing specimen and the detailed information of different layers used in the experiment is illustrated in Table 1.

### 2.3. Thermal Testing

In the experiment, a halogen lamp was used as a heat source. The halogen lamp was placed 300 mm away from the illuminated face of the concrete layer, as demonstrated in Figure 3(c). The power of halogen lamp is 1000 W and the reflectance of illuminated face is 0.47 corresponding to long wave radiation [21]. The illuminated face in the experiment refers to the exterior building surface (outdoor) and the unilluminated surface refers to the interior building surface (indoor). During the experiment, the halogen lamp was powered on and remained constant for 12 hours continuously. The halogen lamp acted as a substitution for radiation heat source in the experiment. Only the outer surface of the specimens was illuminated and the sides of specimens were prevented from heating by reflected radiance. It was noted that there was convective heat transfer from the sides of the specimens. The thickness of specimens is thin so that the area of side surfaces is relatively small compared to that of front surfaces. In addition, the air flow of the lab area where experiments were conducted was slow and the convective heat transfer was minimized. Therefore, the heat conduction through front surfaces was the major part of heat transfer from illuminated panel to unilluminated panel. The temperature of both the illuminated and the unilluminated concrete layers were measured in a one minute interval using the thermocouples embedded at the center of each panel with TDS-303 Data Logger. The measurement range of the equipment is −10°C to 200°C, and the accuracy is ±0.5°C or ±0.5% (whichever is greater). After collecting the temperature data of one specimen, both layers were allowed to cool down without the powered halogen lamp until they reached the ambient air temperature, and the sandwiched layer was then replaced by the other one before starting the next experiment. The illuminated layer and unilluminated layer were repeatedly used in all measurements to make sure that the convective and radiative properties of both layers are consistent throughout the experiments. By observing the temperature variation of both the illuminated and unilluminated layers, the thermal insulating performance among different specimens can be investigated. Furthermore, the temperature observed in experiment is necessary for evaluating the thermal conductivities of concrete and gypsum, which is used for analyzing the heat performance of a three-story building through the finite element approach. This is an important step to link up what has been found from structural element scale to the actual building scale and will be discussed in the next section.

## 3. Finite Element Simulation

In order to investigate the effectiveness of this wall design against the heat transfer across the building envelope, the finite element method (FEM) is adopted by using ABAQUS software for simulating the heat transfer process, including conduction, convection, and radiation, in a three-dimensional three-story building model in which the thermal impact of roof and floors is also considered. During the simulation, different heat conduction properties of materials and nonlinear convection and radiation conditions are considered. Heat transfer can be divided into heat conduction, heat convection, and heat radiation. In real construction, heat exchange with surroundings is mainly through the convection and the radiation, and the heat conduction is the principal factor affecting heat transfer from the exterior to the interior building surfaces. In the simulation, thermal conductivity, density and specific heat capacity of materials are all critical parameters to describe the transient process and the heat conduction process along the building envelope is governed by the following partial differential equation [14]:

(1) $$\begin{array}{c} \rho \cdot c\frac{\partial T}{\partial t}=\frac{\partial }{\partial x}\left(k_{x}\frac{\partial T}{\partial x}\right)+\frac{\partial }{\partial y}\left(k_{y}\frac{\partial T}{\partial y}\right)+\frac{\partial }{\partial z}\left(k_{z}\frac{\partial T}{\partial z}\right)+Q, \end{array}$$

where *T* is the temperature that varies with the time *t* and the position in terms of *x*, *y*, *z* coordinates, *ρ* is the material density, *c* is the specific heat of material, *Q* is the heat source power per unit volume, *k*_*x*_, *k*_*y*_, and *k*_*z*_ are the materials thermal conductivities in *x*, *y*, and *z* directions, respectively. Here, it is assumed that both concrete and gypsum are isotropic medium such that the thermal conductivity (*k*) in all three directions is the same; that is, *k* = *k*_*x*_ = *k*_*y*_ = *k*_*z*_. Two boundary conditions corresponding to the convection and the radiation are required to solve (1), and are shown as follows:

(2) $$\begin{array}{c} -k\frac{\partial T}{\partial n}=h\left(T_{s}-T_{a}\right),\\ -k\frac{\partial T}{\partial n}=\varepsilon \sigma \left(T_{s}^{4}-T_{a}^{4}\right), \end{array}$$

where *n* is the normal vector at surface, *h* is the heat convection coefficient with air, *T*_*s*_ is the temperature at the panel surface, *T*_*a*_ is the ambient air temperature, *ε* is the emissivity coefficient of material, and *σ* is the Stefan-Boltzmann constant which equals 5.67 × 10^−8^ W · m^−2^ · K^−4^.

The material properties play an important role of achieving an accurate prediction of the heat transfer process along the building envelope when solving (1) and (2) using ABAQUS software. Hence, the characterization of the parameters used in the finite element should be carefully conducted. Firstly, it is noted that *ρ*, *c*, and *k* of both concrete and gypsum may change with temperature. However, as the variation of these parameters is minor when the temperature is between 20°C and 70°C, it is assumed that these parameters are independent of temperature in the simulation [22]. Secondly, since the heat conduction is the major part of the heat transfer process along the building envelopes, *k* is one of the most important thermal properties that require a careful evaluation.

### 3.1. Parametric Study

Equipped with the experimental data, the thermal conductivities of both concrete and gypsum can be determined through a parametric study. In the parametric study, two finite element models are constructed based on two types of experimental specimens, namely, C and G, as discussed before. The dimensions of these two models are the same as specimens in the experiment. In this study, a perfect interface contact is assumed, which means the interfaces possess little impact on heat transfer from illuminated layer to unilluminated layer in the simulation. The boundary conditions corresponding to the convection and the radiation are defined on those surfaces which are in contact with air and the air temperatures observed in experiment are imported to both models. Moreover, the load in this parametric study is evaluated in accordance with the power of halogen lamp in the experiment. The thermal conductivities of both concrete and gypsum can be evaluated by varying these two parameters in the FEM until the prediction of the heat performance from the simulation matches with the experimental observation [23]. Some key material properties used in the finite element models are summarized in Table 2 [22, 24].

### 3.2. Simulation of Building Models

**Figure 4:**
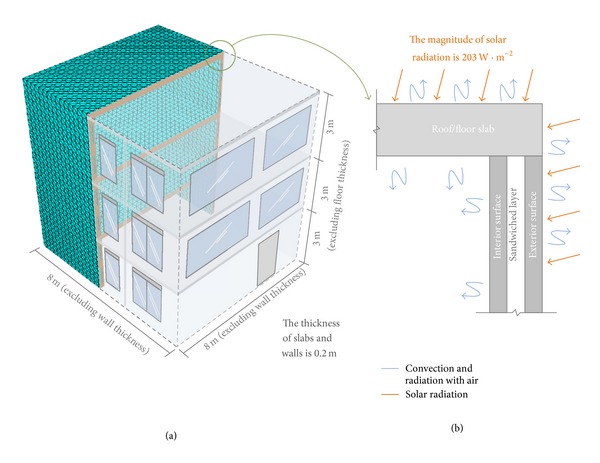
(a)The section view and the dimensions of a three-dimensional three-story building model (with meshing); (b) a two-dimensional schematic diagram showing the boundary conditions in the building model. The solar radiation is defined on exterior surfaces, while the convection and the radiation are defined on all surfaces which are in contact with air, including the exterior and interior surfaces.

After conducting the above parametric study, the required thermal conductivities can be imported into a three-story finite element building model. The section view and the overall dimensions of the model after meshing are shown in Figure 4(a). In these building models, heat transfer through windows and ventilations is not considered. Here, two types of wall panels, namely, C and GV as described in the previous section, are adopted in the building models for the investigation of the heat transfer process in a real building scale (instead of a structural element scale as demonstrated in the experiment). It should be mentioned that the void design of the GV wall panel in the building model is in line with the corresponding experimental specimen, in which the ratio between the void area (*A*_void_) and the entire wall area (*A*_wall_) ranges from 0.2 to 0.4.

The heat source for both building models is solar radiation and the magnitude of solar radiation is varying every single day in reality. In the simulation, average magnitude of solar radiation which equals 203 W · m^−2^ is applied to the outer surfaces of three-story building models [25] and the total illuminating time is assumed to be 12 hours, accompanying no internal heat gains in building models. The initial temperature distributions of both finite element building models are based on the ambient air temperature measured in the experiments. The boundary condition of both models is that all exterior and interior surfaces are in contact with ambient air, including the roof and floors, which is shown in Figure 4(b). Tetrahedral elements are employed to mesh the finite element building models.

## 4. Results and Discussions

### 4.1. Experimental Results

The ambient air temperature of the testing environment was about 24.9°C. The measured temperatures at both the illuminated and the unilluminated layers are summarized in Table 3. The measured temperature at the illuminated layers after 12 hours radiation could reach up to 83.4°C and that at the unilluminated layers in the C, G, and GV specimens were 38.2°C, 36.3°C, and 34.9°C, respectively. By observing the temperature difference between the illuminated and unilluminated layers, the effectiveness of thermal insulation performance of different specimens could be evaluated. As the temperature difference at the C specimen was 1.4°C lower than that at the G specimen, it means the inclusion of a sandwiched gypsum layer in the precast wall panel can effectively improve the thermal insulation capability. In addition, when comparing the G and GV specimens, the temperature difference at the GV specimen was 2.8°C higher than that at the G specimen and it implies that the void inclusion in the sandwiched gypsum layer can further improve the thermal insulation capability. Since the GV specimen is found to be the best for thermal insulation among all the tested specimens, the thermal insulation effect of this new wall panel design is further elucidated through a three-story finite element building model, where the building envelope of this model consists of a sandwiched gypsum layer with voids.

**Figure 5:**
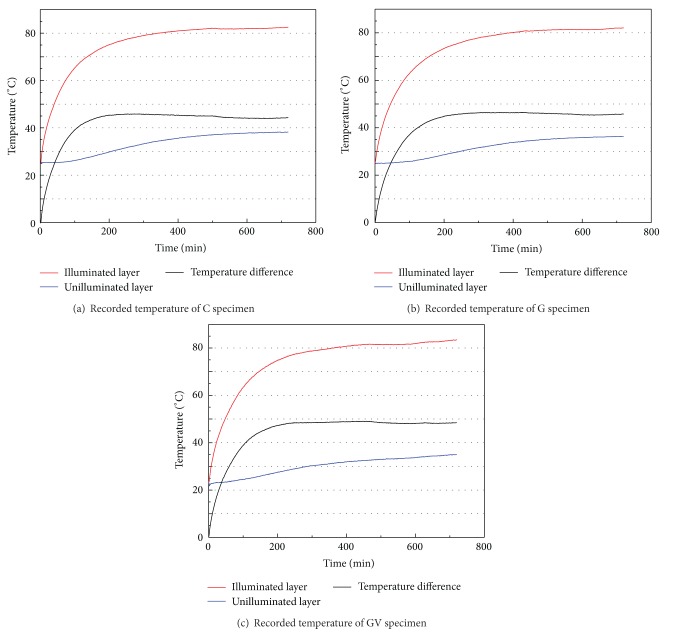
Experimental results showing the temperature variation against time (*T*-*t* curve) in the (a) C specimen, (b) G specimen, and (c) GV specimen.

The recorded temperature variations over time (in the form of *T*-*t* curve) at both the illuminated and the unilluminated layers of all three types of specimen are shown in Figure 5. In the first 200 minutes, the temperature at the illuminated layer of all the specimens increased quickly. After that, the rate of the temperature increase became slower, which implies that the thermal equilibrium between the illuminated layer and the surrounding was gradually approached. Meanwhile, the largest temperature rise of the unilluminated layers happened between 200 to 400 minutes and a thermal balance with the surroundings could be achieved after 600 minutes. This indicates that the thermal input of the unilluminated layers is mainly due to the heat conduction from the illuminated layers. In other words, the experimental results validate the assumption that the conduction governs the heat transfer across the precast wall panel. However, there are some limitations for the experiment. For example, there is heat transfer between unilluminated layer and the foundation of wall panels although the contact area is small. Also, the radiance from halogen lamp cannot replace solar radiation completely. Further work needs to be done to advance the accuracy of experiment.

### 4.2. Results of Parameters Studies

**Figure 6:**
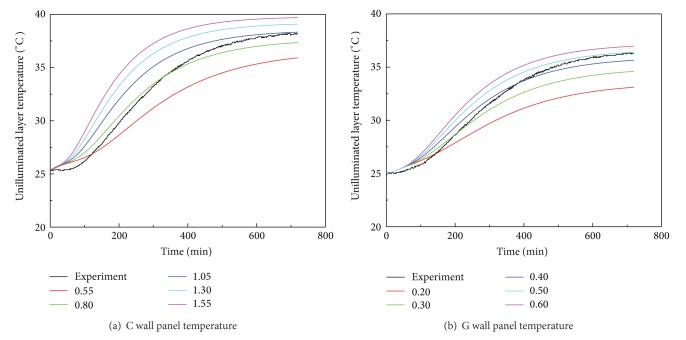
(a) Various *T*-*t* curves of unilluminated layer in C wall panel are generated by varying the thermal conductivity of concrete. The black line refers to the recorded temperature in the C specimen during experiment, while the colored lines are predicted temperature variation corresponding to different thermal conductivities of concrete; (b) various *T*-*t* curves of unilluminated layer in G wall panel are generated by varying the thermal conductivity of gypsum. It is noted that the colored lines are predicted temperature variation corresponding to different thermal conductivities of gypsum.

As the radiation governs the thermal input process in exterior (illuminated) layer of the building envelope and the heat conduction is the main heat absorption process in the interior (unilluminated) layer of the building envelope instead, the characterization of the thermal conductivities of both concrete and gypsum is critical for an accurate evaluation of the heat isolation performance of a three-story building using FEM. The thermal conductivities of both concrete and gypsum are found by a series of parametric studies using the finite element approach. The thermal conductivity of concrete is firstly evaluated by varying this parameter in the FEM representing the C specimen until the prediction matches with the experimental result. In the simulation, the temperature variation against time at the unilluminated layer is monitored and is shown in Figure 6(a). It is observed that the predicted rate of temperature change in the first 200 minutes is higher than the measurement in the associated experiment (i.e., C specimen). This deviation is probably due to the presence of defective interfaces or small air gaps between the contiguous layers in the experimental specimen, whereas the contiguous layers are assumed to contact with each other perfectly in the simulation. As air is a poor conductor, the heat transfer through the interface between two contiguous layers may be retarded with the presence of air. It is shown that the predicted *T*-*t* curve matches well with that from the experiment when the thermal conductivity of concrete equals 1.05 W · m^−1^ · K^−1^ and the relative error between simulated and experimental temperature after 12 hours is less than 3%, which validates the proper selection of 1.05 W · m^−1^ · K^−1^ for concrete thermal conductivity. After evaluating the thermal conductivity of concrete, the conductivity of gypsum can be found using a similar parametric study approach with the aid of the experimental result from the G specimen.  Figure 6(b) shows both the predicted and the experimental *T*-*t* curves at the unilluminated layer in the G specimen. Eventually, the conductivity of gypsum is found to be 0.50 W · m^−1^ · K^−1^, in which the relative error between numerical and experimental temperature after 12 hours is less than 4%. These two key parameters are then employed in the evaluation of the heat flow and temperature distribution in a three-story building structure under solar radiation.

### 4.3. Results and Analysis of Building Simulation

**Figure 7:**
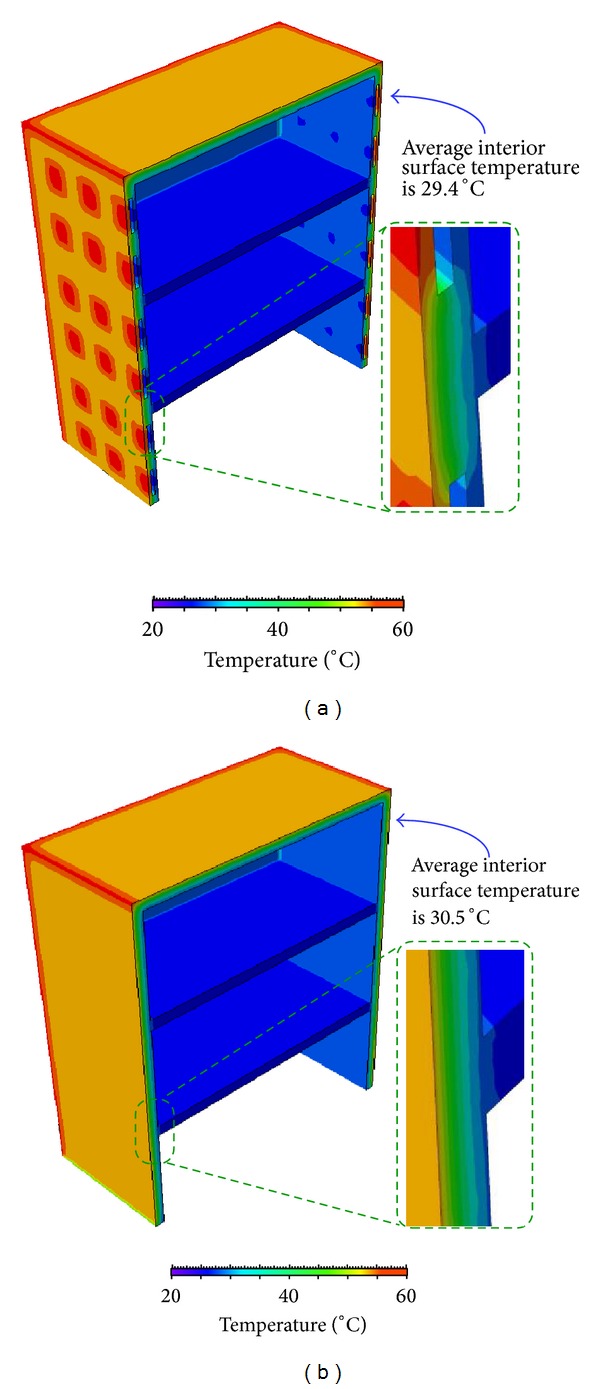
The temperature distribution (after 12-hour solar radiation) of three-story buildings adopting (a) the sandwich concrete/gypsum precast wall panel and (b) the conventional precast concrete wall design. The temperature gradients along the wall in two structures are different. The interior surface temperature of the two buildings is shown and the interior surface temperature is the temperature average measured from all monitoring points.

**Figure 8:**
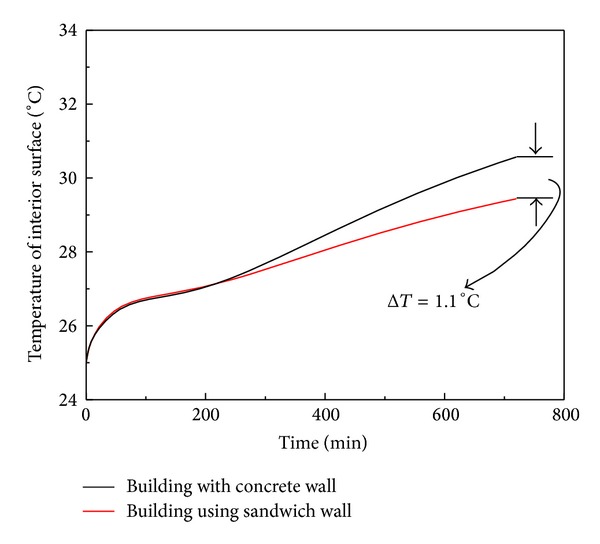
The temperature variations of the interior surfaces in two three-story building models with different wall panel designs.

Two three-dimensional three-story building models using different wall designs (which associate with the C and GV specimens) are constructed for analyzing the temperature distributions along the building envelopes. The contour plots showing the temperature distribution of both models after 12 hours solar radiation are shown in Figure 7.  Figure 7(a) illustrates the temperature distribution of the building using sandwich concrete/gypsum precast wall panel (associated with GV specimen), while Figure 7(b) depicts the temperature distribution of the conventional building with precast concrete wall panel (associated with C specimen). From these contour plots, it is noticed that the temperature of interior surface in sandwich wall design building (29.4°C) is lower than that in conventional design building (30.5°C). In order to monitor the temperature at the interior surface of the building more carefully, abundant monitoring points which are evenly distributed on the interior surface are incorporated in the model to capture the variation of interior surface temperature against time. It should be mentioned that the interior surface temperature of both building models is the average of the temperatures measured from all monitoring points.  Figure 8 shows the interior (unilluminated) surfaces temperature changes against time for both building models under solar radiation for 12 hours. At the beginning, the interior surface temperatures in both buildings are the same. When time goes on, the temperature at the interior surface using the conventional wall panel design (associated with C specimen) is higher than that using the sandwich wall panel design (associated with GV specimen). Meanwhile, it is noticed that the difference of the interior surface temperature between both building models gradually increases with time. After the solar radiation for 12 hours, the magnitude of temperature difference achieves the maximum, that is, 1.1°C, as shown in Figure 8. Although the temperature difference is not huge, this temperature drop with the use of the sandwich wall panel design will lead to a significant reduction of the electricity consumption in the air-conditioning system.

Based on the experimental results, the sandwich wall panel can improve the thermal insulation performance from a structural element scale (i.e., wall element). In addition, the energy saving effect of sandwich wall panel towards an entire building can be further evaluated using the finite element modeling, together with a simple assumption. For simplicity, let us assume that the interior surface temperature is similar to the indoor temperature and the maximum interior surface temperature difference between two buildings is used to predict the energy saving effect of this new wall panel design from a building scale. In order to demonstrate the significance of such temperature drop on the energy saving perspective, two regions in the subtropical area, namely, Texas in US and Hong Kong in China, are chosen as examples. The daylight average temperature in Texas from June 1, 2013 to August 31, 2013 is 34.8°C and that in Hong Kong is 31.1°C [25]. In these two places, the general adopted temperature setting for the air-conditioning system during summer is 20°C. It should be noted that these temperatures are reported by the local governments. By using proposed sandwich concrete/gypsum wall panel, the percentage of energy saving in Texas is 1.1/(34.8 − 20) × 100% = 7.4%, while that in Hong Kong is 1.1/(31.1 − 20) × 100% = 9.9%. Considering the enormous amount of energy consumed by air-conditioning system all over the world, it can be concluded that there is a solid impact on energy saving by this novel wall panel design. In 2009, 3.5 × 10^10^ kWh electrical energy was consumed by air-conditioning in Texas and it accounted for 18% of the total residential electricity consumption [26], while that in Hong Kong was 1.2 × 10^10^ kWh, which accounted for 29% of the total electricity consumption in 2010 [27]. The simulation results indicate that the adoption of sandwich concrete/gypsum wall panel in building structure will lead to (3.5 × 10^10^ kWh × 7.4%)/4 = 6.5 × 10^8^ kWh and (1.2 × 10^10^ kWh × 9.9%)/4 = 3.0 × 10^8^ kWh saving in electricity consumption for air-conditioning system in Texas and Hong Kong, respectively. In fact, this amount of energy saving can fulfill the electricity demand of about 48,000 people per year.

It is reported that the usage of energy gives rise to 83% of global greenhouse gases (GHG) emissions, in which CO_2_ emissions occupy an important proportion in GHG emissions, and the generation of electricity and heat was the major cause of CO_2_ emissions which accounted for 41% of world CO_2_ emissions in 2010 [28]. It is envisioned that the new wall panel design possesses a great potential in reducing CO_2_ emission by the electricity consumed in air-conditioning system. It should be noted that the average emission factors of CO_2_ in Texas and in Hong Kong are 0.5 and 0.7 kg CO_2_ per kWh, respectively, and the difference is because different fuels are used for generating electricity in these two places [28]. Based on the above reported data, it is estimated that 3.3 × 10^8^ kg CO_2_ and 2.1 × 10^8^ kg CO_2_ reduction can be achieved in Texas and in Hong Kong, respectively, by using the new wall panel design. Equipped with this sandwich concrete/gypsum precast wall panel for building construction, sustainable and green building design can be implemented in developed cities through a significant reduction on the buildings energy consumption in air-conditioning system.

## 5. Future Work

It is expected that there may be a drop in mechanical properties of gypsum and concrete/gypsum interface under the effect of prolonged heat and moisture. Further investigation on the durability of this sandwich wall panel should be conducted. More accurate and precise building energy cost simulation can be conducted by some commercial software, such as Energy Plus and Transient System Simulation Tool (TRNSYS), which incorporates the considerations of the building ventilation and climatic effect including daytime temperature, sunshine intensity, and time.

Advanced ceramic thermal coating is another possible approach for a novel design of precast concrete panel with a high insulation against heat. Furthermore, there is an additional measure to reduce heat penetrating into the concrete panel and that is the addition of a thin reflective layer on the external surface of the concrete panel. By an innovative geometric design, it is possible to reflect a proportion of the incident solar radiation back to the sky so that less heat is absorbed by the panel. Such design must prevent any light pollution at the street level. In other words, the current vertical panel design may not be applicable. In the current work, the emphasis is focused on conduction, and it is suggested that various precast concrete panels with different features for the heat insulation against conduction, convection, and radiation should be investigated experimentally.

## 6. Conclusions

Due to the increasing energy demand and the greenhouse effect on the Earth, the buildings energy consumption becomes critical because it is the major cause of CO_2_ emissions. Air-conditioning system is one of the primary sources in the buildings energy consumption, and a considerable amount of energy saving can be obtained by using proper insulating materials or designs to reduce the energy used in air-conditioning system. In this paper, a new sandwich concrete/gypsum wall panel and its application in concrete buildings have been presented. The thermal performance of both the conventional precast concrete wall and the proposed wall panel has been studied by experimental and simulation approaches. Experiments have been performed to validate that the sandwiched gypsum layer can effectively retard the heat transfer process in the precast concrete wall panel and the gypsum layer with voids possesses the greatest thermal insulating capability among the tested specimens. Meanwhile, the thermal conductivities of concrete and gypsum have been carefully evaluated through the parametric studies, as these properties play an important role in the heat transfer process simulation of the building models. In order to interpret the experimental result (which is in a structural element scale) in a real building scale, a comparison of thermal behavior between a building with the sandwich concrete/gypsum wall panel and a conventional concrete building has been investigated by ABAQUS using FEM. It is noticed that the interior surface of the building adopted in the sandwich concrete/gypsum wall panel is 1.1°C lower than that of the convention concrete building, implying that the electricity consumed by air-conditioning system can be saved remarkably when the proposed sandwich concrete/gypsum wall panel is adopted as building envelope. Eventually, a significant reduction in energy consumption and CO_2_ emissions can be achieved.

## Figures and Tables

**Table 1:** Details of the layers used in the tests.

Layers	Specimens	Dimension (mm)			Descriptions
		Length	Width	Height	
Illuminated	—	456	245	65	A thermocouple is embedded at the center; M30 grade concrete
Unilluminated	—	456	245	65	A thermocouple is embedded at the center; M30 grade concrete
Sandwiched	C	456	245	65	M30 grade concrete
	G	436	190	65	A rectangular cube without visible voids
	GV	436	190	65	With 9 voids in a 3 × 3 array Void dimension: 80 × 30 × 65 mm

**Table 2:** Physical properties of the materials at 25°C and 1 atm.

Properties	Concrete	Gypsum
Density (*ρ*) (kg*·*m^−3^)	2300	1500
Specific heat (*c*) (J*·*kg^−1^ *·*K^−1^)	750	1090
Free convection coefficient (*h*) (W*·*m^−2^ *·*K^−1^)	8.9	9.0
Emissivity coefficient (*ε*)	0.85	0.85

**Table 3:** The temperature of specimens after 12 hours radiation.

Specimens	Temperature of the test (°C)			
	Ambient air	Illuminated layer	Unilluminated layer	Difference between two layers
C	25.1	82.5	38.2	44.3
G	25.0	82.0	36.3	45.7
GV	24.5	83.4	34.9	48.5
